# Recurrence of extramedullary plasmacytoma of the breast

**DOI:** 10.3332/ecancer.2013.322

**Published:** 2013-05-28

**Authors:** Khaled Majadob, Wesam Al-Sakkaf, Farouk Rezk, Kris Zegocki, Faris Al-Refaie

**Affiliations:** Haematology Department, The Princess Alexandra Hospital, Harlow, Essex, UK

**Keywords:** extramedullary plasmacytoma, breast plasmacytoma, aggressive plasmacytoma

## Abstract

We present a rare case of a multiple recurrent extramedullary plasmacytoma of the breast and spine that showed aggressive behaviour without bone marrow involvement. A 29-year-old woman initially presented with extramedullary plasmacytoma of the left breast that was treated with radiotherapy. Shortly after, a recurrence appeared in the right breast, spine, and in both breasts, despite treatment with local and systemic therapy.

## Introduction

Extramedullary plasmacytoma (EMP) of the breast is a rare plasma cell neoplasm of the soft tissue that may occur with or without bone marrow involvement or other systemic features of multiple myeloma. Soft tissue extramedullary plasmacytoma (SEP) that occurs as a solitary tumour is extremely rare. SEP commonly involves the soft tissue of the upper respiratory tract, abdomen, mandible, maxilla, and gingiva [1].

In this case report, we present a patient with aggressive recurrent SEP of the breast with a six-month interval between each recurrence. We also aim to describe the response of the patient to the treatment with chemotherapy and radiotherapy at each stage of the disease.

## Case presentation

A 29-year-old woman presented with swelling in the left breast near the nipple as early as October 2009, but this became prominent within one month, and she eventually went on to have fine needle aspiration cytology, which showed plasmacytoid cells in the background, followed by a core biopsy that showed infiltration by immature plasma cells, which were positive for CD138, MUM1, and CD56 and weakly positive for cyclin D1 and EMA. There was lambda light chain restriction. The cells were negative for CD20, CD19, PAX-5, and EBER. They were also negative for MNF 116 and CK7. This supported a diagnosis of plasmacytoma. There was no evidence of any secretory paraprotein in the patient’s blood or urine, and her serum free light chains were normal. An MRI of her spine was clear, as was her bone marrow, which only showed 5% plasma cells. Subsequently, she underwent radical radiotherapy (40.05 Gy in 15 fractions over three weeks).

**Figure 1:**
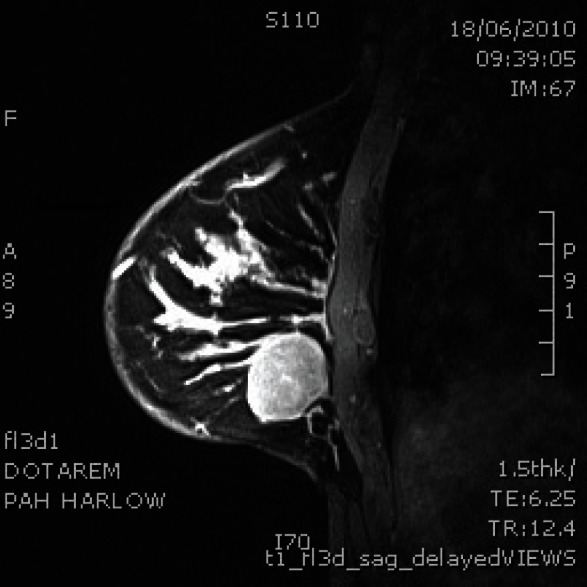
PET scan which shows metabolic activity only in the right breast.

**Figure 2:**
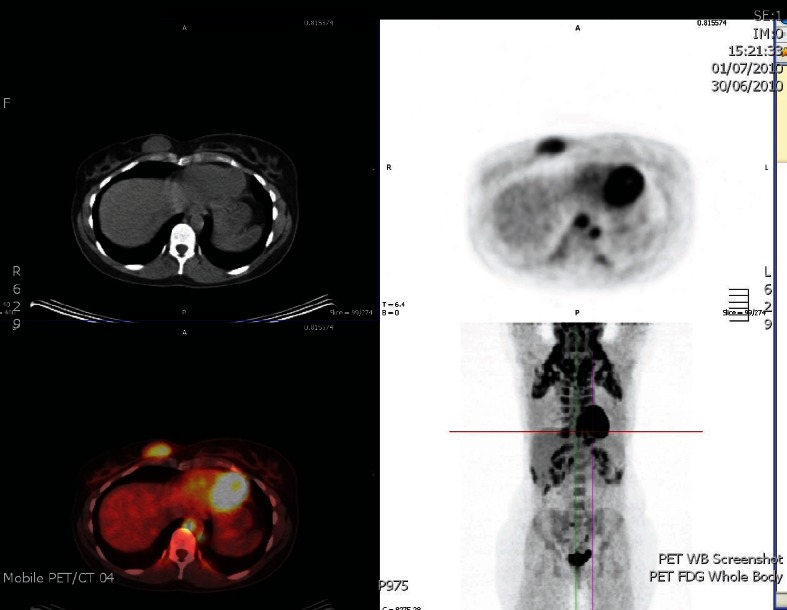
MRI scan of right breast shows well circumscribed solid mass.

Six months later, she re-presented with a lump in her right breast that was 2 cm in size felt clinically, which pointed to a recurrence in the contralateral breast (Figure 1). An ultrasound scan and core biopsy confirmed recurrent disease. Again, investigations, including a PET scan, failed to show myeloma or other lesions. The lump was PET positive (Figure 2). She commenced treatment with CTD (cyclophosphamide, thalidomide, and dexamethasone) in June 2010 and completed five cycles.

**Figure 3:**
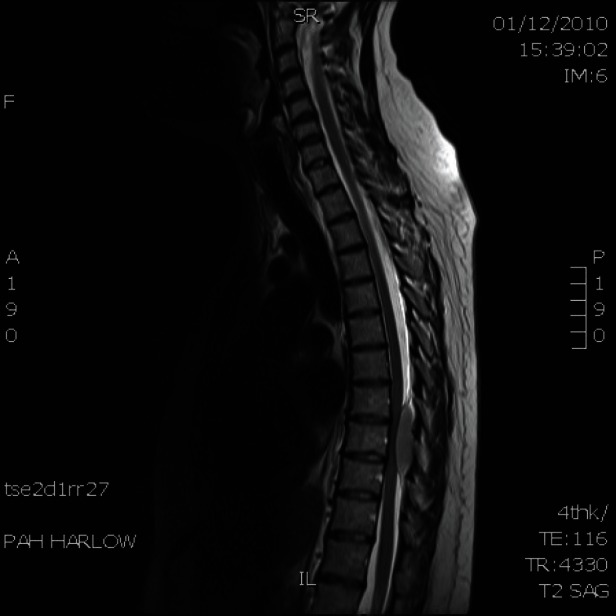
MRI scan of spine shows soft tissue mass posterior to T9 causing moderate cord compression.

Six months later, she presented with increasing back pain when she was due to start her sixth cycle of chemotherapy. She subsequently developed numbness in her feet and loss of power in her legs. An MRI scan showed a mildly enhanced soft tissue mass posterior to the T9, causing moderate cord compression (Figure 3). The patient was treated with urgent radiotherapy (five fractions). She subsequently had another MRI of her spine, which showed resolution of this mass. Her PET scan was clear, as was her bone marrow.

Further chemotherapy (with velcade and dexamethasone) was given, also with consideration for sibling allogenic transplantation.

Three months later, while she was on velcade chemotherapy, she developed a lump in her right breast. A biopsy confirmed recurrent disease. This was the non-irradiated breast. A few days later, she developed two lumps in her left breast (the irradiated breast), although at a different site from her original disease. A biopsy again confirmed recurrence. At this stage, she was treated with PAD (bortezomib, doxorubicin, dexamethasone) followed with an allogenic bone marrow transplant from her HLA-identical match brother. The transplantation was sadly complicated with a neutropenic chest infection that lead to her death.

## Discussion

Breast plasmacytoma is a rare type of EMP, which can occur in the context of multiple myeloma. EMP usually occurs in the head and neck area in 90% of cases, for example, the upper respiratory tract, nasal cavity, paranasal sinuses, oropharynx, and salivary glands [1–3].

The incidence of breast plasmacytomas is very low—only 63 cases were reported between 1928 and 2009. The reported cases were 66% unilateral and 77% associated with myeloma. Regarding these data, the number of reported cases of solitary breast plasmacytoma is about 15–16 cases over the last 80 years [4–12].

Breast plasmacytoma can be misdiagnosed as primary breast cancer. Usually it does not have specific radiological or clinical features [8]. According to 2009 British Committee for Standards in Haematology (BCSH) guidelines, the diagnostic criteria of SEP [13] are as follows:

No M-protein in serum and/or urine

Extramedullary tumour of clonal plasma cells

Normal bone marrow

Normal skeletal survey

No related organ or tissue impairment.

It is well known that plasmacytomas are radiosensitive, with success rates of 79%–90% and a 10-year survival rate of 50%–100% [5–12]. Solitary EMPs have better prognosis than solitary bony plasmacytomas and can be cured by local radiotherapy [5–7].The rate of local recurrence after radiotherapy is less than 5% [14]. In addition, the risk of distant relapse is more than 30%, which is less than that seen in solitary bony plasmacytoma [15].

According to the BCSH guidelines of 2009, recurrent solitary plasmacytoma beyond the original site of radiotherapy, in the continuing absence of systemic disease, may be treated with additional radiotherapy. Patients with more extensive disease or early relapse (as in our patient) may benefit from systemic therapy with or without autologous stem cell transplantation, as indicated for myeloma, with small case series, suggesting long-term disease control. Newer agents including thalidomide and bortezomib have also been used successfully, prior to transplantation, in small numbers of patients with relapsed plasmacytoma.

## Conclusion

Primary breast plasmacytoma usually displays mild clinical behaviour and long patient survival, similar to other plasmacytomas originating from soft tissues [5–6]. The majority of cases can be cured by radiotherapy [7]. Breast plasmacytoma can be misdiagnosed as primary breast cancer. Usually, it does not have specific radiological or clinical features [8]. Careful follow-up is needed as some cases may develop multiple myeloma even after long-term remission [9].
